# Adherence to the South African food based dietary guidelines may reduce breast cancer risk in black South African women: the South African Breast Cancer (SABC) study

**DOI:** 10.1017/S1368980021004675

**Published:** 2022-10

**Authors:** Inarie Jacobs, Christine Taljaard-Krugell, Mariaan Wicks, Jane M Badham, Herbert Cubasch, Maureen Joffe, Ria Laubscher, Isabelle Romieu, Carine Biessy, Marc J Gunter, Sabina Rinaldi, Inge Huybrechts

**Affiliations:** 1 Centre of Excellence for Nutrition, North-West University, Private Bag X6001, Potchefstroom 2520, South Africa; 2 JB Consultancy, Bryanston, Gauteng, South Africa; 3 Department of Surgery, Faculty of Health Sciences, University of Witwatersrand, Houghton, Johannesburg, South Africa; 4 Non-Communicable Diseases Research Division, Wits Health Consortium (PTY) Ltd, Parktown, Johannesburg, South Africa; 5 MRC Developmental Pathways to Health Research Unit, Department of Paediatrics, Faculty of Health Sciences, University of Witwatersrand, Johannesburg, South Africa; 6 Biostatistics Unit, South African Medical Research Council, Tygerberg, Cape Town, South Africa; 7 Centro de Investigación en Salud Poblacional, Instituto Nacional de Salud Pública, Cuernavaca, Morelos, México; 8 Hubert Department of Global Health, Emory University, Atlanta, GA, USA; 9 Nutrition and Metabolism Branch, International Agency for Research on Cancer – WHO, Albert Thomas, Lyon, France

**Keywords:** Breast cancer prevention, Diet and cancer, Dietary guidelines, Black urban women, South Africa

## Abstract

**Objective:**

To determine the level of adherence and to assess the association between higher adherence to the South African food based dietary guidelines (SAFBDG) and breast cancer risk.

**Design:**

Population-based, case–control study (the South African Breast Cancer study) matched on age and demographic settings. Validated questionnaires were used to collect dietary and epidemiological data. To assess adherence to the SAFBDG, a nine-point adherence score (out of eleven guidelines) was developed, using suggested adherence cut-points for scoring each recommendation (0 and 1). When the association between higher adherence to the SAFBDG and breast cancer risk was assessed, data-driven tertiles among controls were used as cut-points for scoring each recommendation (0, 0·5 and 1). OR and 95 % CI were estimated using multivariate logistic regression models.

**Setting:**

Soweto, South Africa.

**Participants:**

Black urban women, 396 breast cancer cases and 396 controls.

**Results:**

After adjusting for potential confounders, higher adherence (>5·0) to the SAFBDG *v.* lower adherence (<3·5) was statistically significantly inversely associated with breast cancer risk overall (OR = 0·56, 95 % CI 0·38, 0·85), among postmenopausal women (OR = 0·64, 95 % CI 0·40, 0·97) as well as for oestrogen-positive breast cancers (OR = 0·51, 95 % CI 0·32, 0·89). Only 32·3 % of cases and 39·1 % of controls adhered to at least half (a score >4·5) of the SAFBDG.

**Conclusions:**

Higher adherence to the SAFBDG may reduce breast cancer risk in this population. The concerning low levels of adherence to the SAFBDG emphasise the need for education campaigns and to create healthy food environments in South Africa to increase adherence to the SAFBDG.

South Africa, an upper-middle income country, is undergoing a rapid nutrition transition characterised by shifts in dietary-and-lifestyle patterns. Nutritious traditional meals and active lifestyles are being replaced with frequent consumption of highly processed foods, often being energy dense and nutrient poor, and high levels of physical inactivity and sedentary behaviour^(1)^. Concurrent to these dietary-and-lifestyle shifts is the continuously increasing rate of obesity, especially among South African women^(2)^. Obesity and overweight are important risk factors for different cancer types, especially postmenopausal breast cancer^(3)^. Breast cancer is the leading diagnosed cancer among South African women and contributes to current public health challenges in South Africa^(4)^. The age-standardised incidence rate of breast cancer in South Africa is 52·6 per 100 000 women, while the age-standardised mortality rate is 16·0 per 100 000 women^(4)^. Incidence rates of breast cancer are lower in black South African women (age-standardised rate of 20·8) compared with other ethnic groups (white, coloured and Indian)^(5)^, but evidence suggests that black women have higher mortality rates compared with other races^(6)^. Based on data from 2020, incidence rates of all cancer cases, including breast cancer, are predicted to rise with 47 % by 2040^(7)^. This is worrisome since higher incidence rates may simultaneously increase mortality rates, especially in resource poor countries such as South Africa^(4)^. It is therefore important to identify specific, modifiable risk factors that could be used to implement preventive actions, especially for black South African women.

The FAO of the UN and WHO recommend country-specific food based dietary guidelines (FBDG) to promote healthy dietary patterns^(8)^. The aim of country-specific FBDG is to reduce nutrition deficiencies and to assist in preventing the development of non-communicable diseases^(8)^. The South African food based dietary guidelines (SAFBDG) were initially published in 2003 and revised in 2012^(9)^. These recommendations consist of eleven short, simple and clear nutrition messages that promote a varied and adequate diet and consider foods that are available, culturally sensitive, affordable and environmentally sustainable^(9)^. However, the level of adherence to the SAFBDG and the association thereof with breast cancer in black women from Soweto are not yet known.

A similar study, conducted in black women from Soweto, South Africa, recently showed that higher adherence to the 2018 World Cancer Research Fund/American Institution for Cancer Research’s (WCRF/AICR) Cancer Prevention Recommendations was associated with a lower breast cancer risk in this population^(10)^. Although the SAFBDG promote similar dietary guidelines as the 2018 WCRF/AICR Cancer prevention guidelines (i.e. increase consumption of fruit, vegetable, beans, lean meats and limit foods high in saturated fat and added sugar), there are some important differences between the two sets of recommendations to consider. Compared with the 2018 WCRF/AICR Cancer Prevention Recommendations, the SAFBDG are guidelines specifically developed for South Africa based on prevailing dietary patterns which aim to address current nutrition-related health problems within South Africa^(9)^. On the other hand, the 2018 WCRF/AICR Cancer Prevention Recommendations are more lifestyle orientated and include cancer-specific recommendations, based on robust evidence from mostly higher income countries^(3)^. Investigating the association between adherence to the SAFBDG and breast cancer risk will complement the previous work of Jacobs and colleagues (2021) by providing additional country-specific or context-specific insight into the diets (and variety thereof) of black women from Soweto, South Africa. The current study aims to first investigate the level of adherence to the SAFBDG (overall and for each individual SAFBDG recommendation) and second to assess whether higher adherence to the SAFBDG (overall and for each individual SAFBDG recommendation) is associated with a reduced breast cancer risk in black South African women from Soweto.

## Methods and study population

The database from the South African Breast Cancer (SABC) study, a population-based, case (*n* 396) control (*n* 396) study conducted among black urban women from the greater Soweto population from 2014 to 2017, was used to conduct the current study. Breast cancer cases were newly diagnosed, prior to any cancer treatment from the Chris Hani Baragwanath Academic Hospital. Cases were recruited as soon as possible after the cancer diagnoses. Controls were healthy and unrelated to the breast cancer cases with no history of cancer diagnoses and matched by age (± 5 years) and area of residence to the cases. Information regarding inclusion and exclusion criteria of breast cancer cases and controls and recruitment of breast cancer cases was previously described elsewhere^(11)^. The sample size had a sufficient power of 80 % (when type II error rate = 10 %) for OR ≥ 1·5 and type I error set at 5 %^(12)^. “This study was conducted according to the guidelines laid down in the Declaration of Helsinki, and all procedures involving research study participants were approved by the International Agency for Research on Cancer, the University of the Witwatersrand and North-West University. Written informed consent was obtained from all subjects/patients.”

### Determining habitual dietary intake

A validated and reproducible culture-specific Quantitative FFQ (QFFQ) was used together with household utensils, food portion pictures and food models to determine habitual dietary intake^(13,14)^. For validation/reproducibility of the QFFQ (in previous studies), spearman rank correlation coefficients of 0·14–0·59 were obtained when macro- and micronutrients intakes and food groups were compared with a 7 d weighed food record and foods captured by a QFFQ^(13,14)^. In 2011l, reproducibility of the QFFQ was evaluated again in the Prospective Urban and Rural Epidemiological-SA study, and correlations for energy, macro- and micronutrients were good (between 0·59 and 0·76), which indicated that the validity of the QFFQ stayed consistent over time^(15)^. The QFFQ was administered by registered dietitians, a nutritionist and registered nurse who all received training by nutritionists with over 30 years of experience in dietary assessment to ensure consistency among the different interviewers. Nutrient content information (macro- and micronutrients) was obtained using the South African Food Composition Tables^(16)^. The dietary intake frequency included the amount of times foods were consumed per d/week/month or never. Life size colour photographs of thirty-seven foods (in three portion sizes) were displayed in the food portion picture booklet. Participants were asked about their habitual dietary intake over the past month. Daily intakes of the different foods included in the QFFQ were calculated by two nutritionists to ensure accurate and quality processing of the QFFQ, using a stepwise approach. A stepwise approach was used to calculate daily intakes. Consumption frequencies were converted into number of days per month, and the amount of each portion consumed (for each individual) was converted into grams, using standardised tables to convert household measurements into grams. The daily consumption was calculated by multiplying the frequency of consumption (d/month) by the portion sizes (converted to grams) divided over 30 d. The daily energy and nutrient intakes were determined by multiplying the daily intake of each food item (as consumed) by the nutrient and energy content (per 100 g), derived from the South African Food Composition tables, and then adding together the contributions from all food items^(16)^.

### Non-dietary assessments

Trained investigators and fieldworkers conducted face-to-face interviews. Detailed information on demographic factors and socio-economic status – ethnicity, history of health, family history of breast cancer, reproductive risk factors (age/year at full-term pregnancy, breastfeeding history, age at menarche and at menopause for postmenopausal women, use of oral contraceptives and hormone replacement therapy), family history of cancer, breast health (previous breast lumps by breast laterality and breast pains), smoking habits and physical activity (recreational, transportation, occupational and household) – were collected. Weight, height, sitting height, hip circumference and waist circumference were measured according to standardised procedures. Measurements were always done in duplicate and redone if there was discordance. Measurements were done by the same person throughout the study to avoid inter-user variation, and all equipment was calibrated regularly. All questionnaires used to collect the anthropometry and lifestyle information were validated and proven reproducible in studies conducted in South Africa and elsewhere^(17,18)^.

### Construction of the South African food based dietary guidelines adherence score

#### To determine the level of adherence to the South African food based dietary guidelines (overall and for each individual South African food based dietary guideline recommendation)

To determine the level of adherence to the SAFBDG, an adherence score was developed since no standardised scoring algorithm currently exists to measure adherence to the SAFBDG. Table 1 provides a detailed layout of the construction of the adherence score with recommended intakes/adherence levels, methods and definitions used for each recommendation to determine adherence to the SAFBDG. Each of the participants’ adherence to the SAFBDG was calculated for overall adherence as well as for each individual SAFBDG recommendation. Recommendations regarding salt (use salt and foods high in salt sparingly) and water (drink lots of clean, safe water) consumption were excluded from the overall adherence score as the required information to assess adherence to these recommendations were not gathered during data collection.

**Table 1:** A detailed layout of suggested food intakes, methods used and foods included in each food groups to construct food groups for measuring adherence to the South African food based dietary guidelines (SAFBDG)

Recommendation	Suggested intake/measure of adherence	Method used to measure adherence to the individual SAFBDG recommendation	Foods included in food groups to measure adherence to the guideline/Exclusions and definitions
1) Enjoy a variety of foods	DDS > 4 (out of 9) A DDS score <4 was considered a low dietary diversity.	Calculating a DDS with nine food groups, using the FAO guidelines, with nine being the highest DDS and zero (0) the lowest DDS^(28)^.	1·1) Cereals, roots and tubers: rice, corn, sorghum, oats, samp, sweet potatoes, potatoes, fortified and unfortified maize meal and bread, products made with wheat flour or any other grain flour such as pasta, porridges and tortillas Excluded: sweet biscuits and cakes as they are classified under “sweets” (not measured as part of the DDS in the current study).
			1·2) Meat, poultry and fish: organ meat, chicken (fresh and frozen), fish and seafood (fresh, frozen and canned), red meat (beef, pork, goat, mutton, venison), processed meat (salami, sausage/vienna, ham, polony, corned beef, bacon) and dried meat (biltong, mopani worms).
			1·3) Dairy: milk (whole, low-fat and skimmed milk) and milk products (custard, hard and soft cheese, processed cheese and yoghurt).
			1·4) Eggs: scrambled, fried and boiled/poached.
			1·5) Vitamin A-rich fruit and vegetables (for plant foods – 60 RAE per 100 g & for liquids- 30 RAE per 100 g)^(28)^: carrots, pumpkin, red pepper, sweet potato (yellow flesh), spinach, apricots, melon (orange flesh), mango, pawpaw, apricot juice and peaches. Vegetables mixed with potatoes were separated according to standardised recipes from the Condensed Food Composition Tables of South Africa.
			1·6) Legumes/pulses: beans (dried, cooked, raw and canned), peanuts, peanut butter, lentils and soya products.
			1·7) Other fruit: banana, fig, grapes, guava, raisins, naartjie/tangerine, orange, blueberry, plum, strawberry, melon (green flesh), watermelon, pineapple, pear, apple, litchi, pomegranate, avocado and kiwifruit.
			1·8) Other vegetables: beetroot, onions, broccoli, cauliflower, lettuce, cabbage, green beans, brinjal, cucumber, mushroom, tomato, gem squash, peppers and okra. Mixed vegetable dishes were separated according to standardised recipes from the Condensed Food Composition Tables of South Africa.
			1·9) Fats and oils: butter, tallow, margarine, mayonnaise, vegetable oil, shortenings and sour cream.
2) Be active.	At least 150 min of moderate and vigorous physical activity/week^(19)^	Calculating the sum of light, moderate and vigorous physical activity (min/week).	Due to low levels of moderate and vigorous physical activities in both breast cancer cases and controls, light physical activities were included in the analysis.
3) Make starchy food part of most meals*	No specific suggestion. General guideline of ten minimally processed/whole grains/root vegetables exchanges/units/day (based on 8 500 kJ intake/d)^(27)^.	Exchange quantities for one unit: + maize meal porridge, + soft/maltabella/oats = 125 g + maize meal porridge, stiff = 60 g + maize meal porridge, crumbly = 45 g + bread = 35 g + potatoes/sweet potatoes = 100 g +cooked, pasta/samp/whole grains = 75 g, +unsweetened breakfast cereals = 25 g + cooked rice = 65 g	Included: carbohydrates in the form of minimally/unprocessed or whole grains and root vegetables such as potatoes and white fleshed sweet (roasted, boiled, mashed, sautéed in sunflower oils, no added sugar or saturated fats) potatoes, fortified white and brown bread, whole wheat bread, fortified and unfortified maize meal, white and brown rice, plain pasta (macaroni, spaghetti), sorghum, bulgur wheat, oats and minimally processed breakfast cereals without added sugar (see definition of added sugar in table below). Excluded: highly processed/refined starchy foods (provita’s, biscuits, cookies, cakes, tarts, pies), deep fried potatoes/French fries, vetkoek (deep fried dough) and legumes (legumes are included in the individual SAFBDG recommendation “Eat dry beans, split peas, lentils and soya regularly”).
4) Eat plenty of vegetables and fruit every day.	5 portions or 400 g/d^(21)^.	Calculating the sum of all fruit and vegetables consumed in g/d, using the food composition tables of South Africa^(16)^	Vegetables: spinach, green beans, green leafy vegetables, beetroot, carrot, onions, broccoli, cauliflower, lettuce, cabbage, brinjal, baby marrow, okra, cucumber, corn, mushrooms, tomato, gem squash, green pepper. Excluded: potato and white fleshed sweet potato (included in the individual SAFBDG recommendation “Make starchy foods part of most meals”.
			Fruit: banana, apple, apricot, melon (orange and green flesh), figs, guava, raisins, mango, tangerines/naartjies, orange, pawpaw, peach, blueberry, peach, plum, strawberry, watermelon, pineapple, pear, fruit salad, prune, litchi, pomegranate, avocado, kiwifruit and 100 % pure fruit juice.
5) Eat dry beans, split peas, lentils and soya regularly†	No specific indication. At least 2 to 3 portions/week. One portion equals ½ cup or 75 g of dry beans/peas/cooked lentils or 30 g of soya mince.	Calculating the sum of all legumes consumed in g/d, using the food composition tables of South Africa^(16)^.	Beans, split peas, lentils (dried, canned, raw and cooked) and soya products.
6) Have milk, maas or yoghurt every day.	Milk, maas or yoghurt at least >400 ml/d^(26)^.	Calculating the sum of milk, maas or yoghurt in ml/d, using the food composition tables and food quantities table of South Africa^(16,48)^.	Milk (either fresh or powdered), unsweetened yoghurt and maas (traditional fermented milk). Excluded: sweetened, full-fat and processed milk products and cheeses to prevent intake of SFA, Na and sugar^(35)^.
7) Fish, chicken, lean meats or eggs can be eaten daily.	2 to 3 fish servings/week Approximately 4 Eggs/week Lean meat ≤90 g/d^(22)^	Calculating the sum of fish, chicken, lean meats and eggs consumed in g/d, using the food composition tables of South Africa^(16)^	Fish and seafood, especially oily fish such as sardines, pilchards, tuna, anchovies and mackerel (fresh, frozen and canned), chicken (white and dark meat), lean meat and eggs (scrambled, fried, boiled/poached). Lean meat is defined as meat with a fat percentage between 5 % and 10 % can be labelled as lean or trimmed^(46)^.
8) Use fats sparingly. Choose vegetable oils rather than hard fats.	≥20 % Total fat intake ≤30 % of total energy intake/d. Saturated fat <10 % of total energy intake/d^(24)^.	Using the total amount of fat as a percentage of total energy intake and total amount of saturated fat as a percentage of total energy intake. Measured by the food composition tables of South Africa^(16)^	Total fat measured in all single foods consumed in breast cancer cases and controls. Total fat includes trans fatty acids, SFA, MUFA and PUFA (including essential fatty acids such as *n*-3, *n*-6). Hard fats refer to all animal fats and vegetables oils such as palm kernel and coconut oil.
9) Use sugar and foods and drinks high in sugar sparingly.	Added sugar intake <10 % of total energy intake. Added sugar intake <6 % for further health benefits^(25)^.	Total amount of added sugar as a percentage of total energy intake, measured by the food composition tables of South Africa^(16)^	Added sugar measured in all single foods consumed in breast cancer cases and controls. ‡Added sugar refer to “any sugar added to foodstuffs during processing and includes but is not restricted to sugar as defined by Regulations Relating to the Use of Sweeteners in Foodstuffs under the Act, honey, molasses, sucrose with added molasses, coloured sugar, fruit juice concentrate, deflavoured and/or deionized fruit juice and concentrates thereof, high-fructose corn syrup and malt or any other syrup of various origins”^(47)^.

DDS, dietary diversity score; RAE, retinol activity equivalent.

* No specific indication of portion size or frequency of consumption by the SAFBDG. General guideline is to consume ten starchy food guide units per day (based on 8500 kJ intake/d)^(27)^ (one food guide unit: maize meal porridge, soft/maltabella/oats = 125 g, maize meal porridge, stiff = 60 g, crumbly = 45g bread = 35 g, potatoes/sweet potatoes = 100 g, cooked pasta/samp/whole grains = 75 g, unsweetened breakfast cereals 25 g and cooked rice = 65 g) of the study population^(48–50)^.

† To establish the recommended portion size to measure adherence to this guideline, legume consumption was compared with the legume consumption in other South African studies (PURE, SANHANES), and guidance from the Nutrition Information Centre of the University of Stellenbosch was followed^(29–31)^.

‡ Definition of added sugar as described in the Regulations Relating to the Labelling and Advertising of Foodstuffs, No. R. 146 of 1 March 2010. Foodstuffs, Cosmetics and Disinfectants Act (Act 54 of 1972)^(47)^.

A maximum overall adherence score of nine (to the SAFBDG) was therefore possible. To determine the level of adherence to the SAFBDG, overall and for each individual SAFBDG recommendation, suggested portions sizes/d or week and percentages of total energy intake per day were used as cut-points (as currently suggested in the technical support papers of the SAFBDG)^(19–27)^. As an example, it is advised to consume 400 g fruit and vegetables and to have <10 % of total energy from saturated fat intake/d^(21,24)^.

To calculate adherence to the individual SAFBDG recommendation “Enjoy a variety of foods”, a Dietary Diversity Score (DDS), based on nine food groups, was used. The nine food groups were based on the FAO of the UN guidelines and included (1) cereals, roots and tubers; (2) meat, poultry and fish; (3) diary; (4) eggs; (5) vitamin A-rich vegetables and fruit; (6) legumes; (7) other vegetables than vitamin A-rich; (8) other fruits than vitamin A-rich and (9) fats and oils^(28)^. The lowest possible DDS score was zero (0) and the highest possible DDS nine (9). A DDS score below four (4) was considered as having a low dietary diversity^(28)^.

No specific portion size was stated in the SAFBDG to measure adherence to the individual SAFBDG recommendation “Make starchy foods part of most meals”^(27)^. To assess adherence to this recommendation, a general guideline (consume ten starchy exchanges/units/d, based on 8500 kJ intake/d) was used^(37)^. Starchy exchanges/units included minimally processed starches, whole grains and root vegetables (potato, white fleshed sweet potato)^(27)^. With regard to measuring adherence to the SAFBDG recommendation “Eat dry beans, spilt peas, lentils and soya regularly”, no specific amount (percentage of total energy) or portion size was stated in the SAFBDG^(19)^. To establish the recommended portion size to measure adherence to this guideline, legume consumption was compared with the legume consumption in other South African studies (Prospective Urban and Rural Epidemiologica, SANHANES) and guidance from the Nutrition Information Centre of the University of Stellenbosch was followed^(29–31)^. Sensitivity analysis was conducted without the recommendation “Eat dry beans, spilt peas, lentils and soya regularly” and “Make starchy foods part of most meals” but did not change the overall adherence result (results not shown).

Adherence scores to each individual SAFBDG recommendation, using recommended portion sizes, were calculated as follows: one (1) point when adhering to the recommendation and zero (0) points for non-adherence to the recommendation. Two sub-categories were developed to measure adherence to the recommendations “Fish, chicken, lean meat or eggs can be eaten daily” and “Use fats sparingly. The two sub-categories for the recommendation “Fish, chicken, lean meat or eggs can be eaten daily” included: (1) fish, chicken and lean meat consumption can be eaten daily and (2) egg consumption can be eaten daily. The two sub-categories to measure adherence to the recommendation “Choose vegetable oils rather than hard fats” included (1) keep total fat intake between the recommended range (≥20 % and ≤30 % of total energy intake) and (2) limit saturated fat intake <10 % of total energy intake. Adherence to these individual SAFBDG recommendations with two sub-categories, mentioned above, was scored as follows: zero (0) points for non-adherence and half a point (0·5) for adherence. To measure adherence to the individual SAFBDG recommendation “Use sugar & food & drinks high in sugar sparingly”, three sub-categories, non-adherence (0 points), partial adherence (0·5 point) and adherence (1 point) were developed. This was based on the fact that the SAFBDG, in accordance with the WHO’s healthy diet indicator, advise an added sugar intake of <10 % of total energy intake, while an added sugar intake of <6 % of total energy may have additional health benefits^(25)^.

#### Assessing the association between adherence to the South African food based dietary guidelines (overall and for each individual South African food based dietary guideline recommendation) and breast cancer risk

The distribution of adherence to five (out of nine) of the individual SAFBDG recommendations resulted in highly skewed categories (adherence/non-adherence to an individual SAFBDG recommendation ≥73 % for both breast cancer cases and controls). Therefore, data-driven tertiles (33rd and 66th percentiles) were used to determine the cut-off points for assessing the association between adherence to the SAFBDG (overall and for each individual SAFBDG recommendation) and breast cancer risk. Cut-off points for each individual SAFBDG recommendation, using data-driven tertiles, were calculated as follows: one (1) point when adhering to the recommendation (highest tertile); half (0·5) a point for partial adherence to the recommendation (middle tertile) and zero (0) points for non-adherence to the recommendation (lowest tertile) (see online Supplemental Table 1). Each individual SAFBDG recommendation contributed equally to the total adherence score. The recommendations, “Use fats sparingly” and “Choose vegetable oils rather than hard fats” had two sub-recommendations that were scored individually and were divided by two to determine an average score (0; 0·25 and 0·5). Finally, tertiles of control participants (33rd and 66th percentiles) were used to determine adherence to the overall score and the association with breast cancer risk, with ≤3·5 being the lowest adherence tertile and >5·0 the highest adherence tertile.

### Statistical analysis

A total of 399 breast cancer cases and 399 matched controls were recruited in the SABC study. Of those, three breast cancer cases and three matched controls were excluded due to missing dietary data information. Descriptive analyses were performed, and differences between cases and controls were assessed using paired sample t-test (normal distributed data presented as mean ± s d) and Wilcoxon signed rank test (not normal data, presented as median, 25th and 75th percentiles) for continuous variables and paired *χ*^2^ test for categorical variables (presented as percentages). Specifications of the WHO were used to calculate BMI, using measured height and weight (kg/m^2^).

### Assessing the association between adherence to the South African food based dietary guidelines and breast cancer risk: overall and individual guidelines

Conditional logistic regression models were used to compute OR and associated 95 % CI to determine the association between breast cancer risk and adherence to the SAFBDG (overall and each individual SAFBDG recommendation). Adherence scores (overall and for each individual SAFBDG recommendation) were stratified by hormonal breast cancer receptor subtypes, menopausal status (pre *v*. post) and obesity (BMI ≥ 30 kg/m^2^). For the two latest variables, unconditional logistic regression was used. Additional analysis was conducted to determine significant interactions among strata (menopausal status, hormonal breast cancer receptors and obesity).

The following confounders were examined for adherence to the overall- and individual SAFBDG recommendation (chosen a priory from known breast cancer risk factors): individual income (R1–R3000, R3001–R6000 and R6001+), ethnicity (Zulu/Pedi/Swazi, Xhosa, Sotho, Tshwane, Venda, Tsonga and Ndebele), level of education (none/primary school, high school and college/postgraduate/diploma), smoking (smokers and non-smokers), height (continuous), waist circumference (continuous data), age at menarche (continuous), full-term pregnancy (yes/no), age at first pregnancy (<24 *v*. >24 years of age), age at menopause (<48 *v*. >48 years of age), parity (≤ three children *v*. > three children), duration of exclusive breast-feeding (months), use of exogenous hormones (hormonal birth control to avoid pregnancy: oral contraceptives and injections), or hormone replacement therapy after menopause), family history of breast cancer (yes/no), HIV status (positive *v*. negative), total energy intake in kJ (continuous), alcohol intake in gram (continuous) and under reporting (under reporting, plausible reporting and over reporting). Under reporting (13·1 % of breast cancer cases and 11·6 % of controls) and over reporting (24·0 % of breast cancer cases and 27 % of controls) cut-off points were calculated using the Goldberg and Black principle to determine over-and-under reporting of energy intake^(32)^. Menopausal status, ethnicity, total energy intake, alcohol intake, individual income/month and waist circumference altered the crude OR by more than 10 % when assessing adherence to overall and individual SAFBDG recommendations and were included in our final model.

An additional confounder, habitual physical activity per day (active *v*. less active), was examined when adherence to the individual SAFBDG recommendations was assessed. This was done as physical activity was part of the overall score and as such not included as a confounder in the overall score analyses.

## Results

Selected descriptive characteristics amongst cases and controls are reported in Table 2. Ethnicity differed significantly between cases and controls with breast cancer cases having more Ndebele-speaking people and controls having more Sotho-speaking people. Breast cancer cases had a significant lower waist circumference (93·3 cm ± 13·8 cm) compared with controls (95·8 cm ± 13·7 cm) and had less HIV-positive (16·5 %) cases than controls (22·6 %). Controls had a higher percentage of alcohol consumers but consumed less ethanol (4·6 g/d) on average than cases (5·4 g). Hormone-responsive breast cancers, ER+ (75·3 %) and PR+ (66·4 %) were the dominant breast cancer subtypes, while triple negative breast cancer accounted for 16·2 % (not stratified by menopausal status).

**Table 2:** Distribution of characteristics between breast cancer case and control participants (means ± sd, median and 25th; 75th percentiles, based on distribution of variables)

Characteristics	Breast cancer cases (*n* 396)		Controls (*n* 396)		*P*-value
	*n*	%	*n*	%	
Socio-demographic factors					
*Age (years)					
Mean	54·7		54·6		0·980
sd	12·9		12·9		
Ethnicity					0·041
Zulu/Pedi/Xhosa/Tswana/Swazi	67	16·9	66	16·6	
Sotho	108	27·3	144	36·4	
Venda/Tsonga	105	26·5	91	23·0	
Ndebele	116	29·3	95	24·0	
Level of education					0·078
None/primary	97	24·5	71	17·9	
High School	257	64·9	279	70·5	
College/university/postgraduate	42	10·6	46	11·6	
Individual income/month					0·350
R0	125	31·6	108	27·3	
R1–R3000	219	55·3	227	57·3	
R3001–R6000+	52	13·1	61	15·4	
Anthropometry					
*BMI (kg/m^2^)					
Mean	31·4		31·8		0·317
sd	7·0		6·9		
*WC (cm)					
Mean	93·3		95·8		0·011
sd	13·8		13·7		
Lifestyle factors					
†Total vigorous and moderate PA min/week					
Median	39·4		32·1		0·303
25th percentile, 75th percentile	7·8, 85·8		9·1, 70·8		
Current smokers	35	8·8	44	11·1	0·286
HIV positivity	65	16·4	90	22·7	0·025
Dietary factors					
†TE (kJ/d)					
Median	9146		8990		0·239
25th percentile, 75th percentile	6812, 9759		7184, 10 284		
†Protein (g/d)					
Median	63·8		63·5		0·073
25th percentile, 75th percentile	47·4, 82·7		49·2, 93·1		
% of TE	11·8		12·0		
†Total fat (g/d)					
Median	64·8		64·4		0·125
25th percentile, 75th percentile	42·4, 91·9		47·2, 95·7		
% of TE	26·9		27·2		
†Saturated fat (g/d)					
Median	17·9		19·2		0·044
25th percentile, 75th percentile	11·5, 26·1		12·7, 27·9		
% of TE	7·4		8·1		
*CHO (g/d)					
Mean	330·8		338·7		0·445
sd	143·5		147·3		
% of TE	61·4		64·0		
*Dietary fibre (g/d)					
Mean	24·9		25·3		0·616
sd	11·03		11·4		
†Added sugar (g/d)					
Median	65·3		67·9		0·313
25th percentile, 75th percentile	38·4, 105·5		39·9, 109·7		
% of TE	12·1		12·0		
Non-alcohol consumers	350	88·4	321	81·1	0·004
†Ethanol intake in alcohol consumers (g/d)					
Median	5·4		4·6		0·005
25th percentile, 75th percentile	2·8, 13·8		2·5, 14·7		
†Dietary diversity score					
Median	3·0		4·0		<0·001
25th percentile, 75th percentile	2·0, 4·5		2·5, 7·0		
*Starchy staple foods (g/d)					
Mean	543·1		574·5		0·095
sd	270·0		258·8		
†Fruit, fruit juice and non-starchy vegetables (g/d)					
Median	342·5		418·3		0·001
25th percentile, 75th percentile	169·1, 627·3		208·9, 1098·1		
†Legumes (g/d)					
Median	14·3		18·7		0·171
25th percentile, 75th percentile	6·0, 49·6		6·0, 101·1		
†Milk, maas or yoghurt (g/week)					
Median	91·7		100·0		0·004
25th percentile, 75th percentile	22·9, 141·5		43·1, 195·0		
†Fish, chicken, lean meat or eggs (g/d)					
Median	43·9		56·8		0·061
25th percentile, 75th percentile	17·1, 100·0		21·4, 145·5		
Breast cancer risk factors					
Full-term pregnancy in parous women	377	95·2	382	96·5	0·374
Ever breast fed in parous women	339	91·4	349	89·9	0·496
†,‡Duration of breast-feeding (months)					
Median	35		41		0·187
25th percentile, 75th percentile	20, 62		24, 62		
§Premenopausal	133	33·6	134	33·8	0·852
§Postmenopausal	248	65·1	257	65·7	0·852
†Age at menarche					
Median	15		15		0·537
25th percentile, 75th percentile	13, 16		13, 16		
†,||Age at menopause (years)					
Median	47		48		0·331
25th percentile, 75th percentile	42, 50		44, 50		
Family history of breast cancer	25	6·3	17	4·3	0·205
Use of birth control (contraceptives)	229	57·8	215	54·3	0·238
Breast cancer case characteristics					
Receptor status					
ER+	298	75·3	–		
PR+	263	66·4	–		
HER2	114	28·8	–		
¶Breast Cancer case subtype					
HER2 enriched	21	5·3	–		
Luminal A	40	10·1	–		
Luminal B	269	67·9	–		
TNBC	64	16·2	–		

WC, waist circumference; TE, total energy; CHO, carbohydrates; PA, physical activity; ER+, oestrogen receptor positive; PR+ progesterone receptor positive; HER2, Human-Epidermal Growth Factor-2; TNBC, triple negative breast cancer; HRT, hormone replacement therapy.

* Data are presented as means and standard deviations (sd).

† Data are presented as median (25th percentile, 75th percentile).

‡ In breast feeding women only.

§ Twenty missing values for menopausal status (fifteen cases and five controls) Missing values were excluded from percentage calculations.

|| Among postmenopausal women only.

¶ Defined using Allred scores.

Table 3 presents the level of adherence to the SAFBDG (overall and for each individual SAFBDG recommendation) between cases and controls, using suggested portion sizes or percentages of total energy intake as cut-points for all the SAFBDG recommendations. Regarding overall adherence to the SAFBDG, only 32·3 % of breast cancer cases and 39·1 % of controls adhered to at least half (4·5) of the SAFBDG (nine out of the eleven were measured).

**Table 3:** Measuring the level of adherence to the South African food based dietary guidelines (SAFBDG) between breast cancer cases and controls, using recommended portion sizes or percentages of total energy intake

SAFBDG recommendations	Recommended portion or % of TEI for a healthy diet	Score criteria	Cases *n*	%	Controls *n*	%	*P*-value
1) Enjoy a variety of foods	DDS score <4	0	241	60·9	194	48·9	0·001
	DDS score ≥4	1	155	39·1	202	51·1	
2) Be active	Moderate PA < 150 min/week	0	395	99·7	393	99·2	0·563
	Moderate PA ≥ 150 min/week	1	1	0·3	2	0·8	
3) *Make starchy foods part of most meals	Less than ten starch exchanges/d	0	247	62·4	228	57·6	0·168
	At least ten starch exchanges/d	1	149	37·6	168	42·4	
4) Eat plenty of vegetables and fruit everyday	Fruit & vegetables <400 g/d	0	221	55·8	196	49·5	0·075
	Fruit & vegetables ≥400 g/d	1	175	44·2	200	50·5	
5) †Eat dry beans, split peas and lentils regularly	‡Less than 2 or 3 times/week	0	328	82·8	306	77·3	0·050
	‡At least 2 or 3 times/week	1	68	17·2	90	22·7	
6) Have milk, maas or yoghurt everyday	Milk, maas or yoghurt <400 ml/d	0	391	98·7	390	98·5	0·761
	Milk, maas or yoghurt ≥400 ml/d	1	5	1·3	6	1·5	
7) Fish chicken, lean meat or eggs can be eaten daily‖	Lean meat or fish >90 g/d	0	71	17·9	107	27·0	0·002
	Lean meat or fish ≤90 g/d	0·5	325	82·1	289	73·0	
	§Eggs <4/week	0	241	60·8	211	53·3	0·031
	§Eggs ≥4/week	0·5	155	39·2	185	46·7	
8) Drink lots of clean safe water	Not included for analysis						
9) Use fats sparingly. Choose vegetable oils rather than hard fats (divided into two subgroups)‖	Total fat <20 % or >30 %	0	188	47·5	217	54·8	0·039
	Total fat ≥20 % & ≤30 %	0·5	208	52·5	179	45·2	
	Saturated fat ≥10 % of TEI	0	323	81·6	318	80·3	0·651
	Saturated fat <10 % of TEI	0·5	73	18·4	78	19·7	
10) Use sugar & foods & drinks sparingly¶	Added sugar ≥10 % of TEI	0	256	64·6	248	62·6	0·719
	Added sugar <10 % & ≥5 % of TEI	0·5	97	24·5	98	24·7	
	Added sugar <6 % of TEI	1	43	10·9	50	12·7	
11) Use salt & food high in salt sparingly	Not included for analysis						
Overall adherence score							
Total adherence score							
Median			4		4		
25th and 75th percentiles			2·75, 5·0		3·0, 5·75		
Adherence score >4·5			128	32·3	155	39·1	0·045

SAFBDG, South African food based dietary guidelines; TEI, total energy intake; DDS, dietary diversity score.

* No specific indication of portion size or frequency of consumption by the SAFBDG. General guideline is to consume ten starchy food guide units per day (based on 8500 kJ intake/d)^(27)^ (one food guide unit: maize meal porridge, soft/maltabella/oats = 125 g, maize meal porridge, stiff = 60 g, crumbly=45g bread=35 g, potatoes/sweet potatoes = 100 g, cooked pasta/samp/whole grains = 75 g, unsweetened breakfast cereals 25 g and cooked rice = 65 g) of the study population^(49,50)^.

† No specific indication of portion size or frequency of consumption by the WHO or SAFBDG. Current frequency is based on estimate recommendation from global food based dietary guidelines and national recommendations^(30,50)^.

‡ One serving equals 75 g of dry beans, peas, cooked lentils or 30 g of soya mince or 21·4 g/d^(50)^.

§ The current recommendation suggest up to 4 eggs/week and equals 29 g/d (based on the weight of one large egg, 50 g).

‖ Guideline is divided into two subgroups.

¶ Guideline has three categories to measure adherence.

Both cases and controls showed adherence levels <50 % to the following individual SAFBDG recommendations: “Be active”, “Make Starchy foods part of most meals”, “Eat plenty of fruit and vegetables every day”, “Eat dry beans, spilt peas and lentils regularly”, “Have milk, maas or yoghurt every day” and “Use sugar & foods & drinks sparingly”. Fewer cases adhered to the individual SAFBDG recommendation “Enjoy a variety of foods” (measured by a DDS score), with only 39·1 % of cases having a dietary diversity score above four (out of nine) compared with 51·1 % of controls. Both cases (17·2 %) and controls (22·7 %) showed low adherence to the individual SAFBDG recommendation “Eat dry beans, spilt peas and lentils regularly”. In addition, both cases (82·1 %) and controls (73 %) showed high adherence to the sub-category for fish, chicken and lean meat consumption (<90 g/d), while controls (46·7 %) were more likely to adhere to the sub-category on egg consumption (at least four eggs/week) than breast cancer cases (39·2 %). Although adherence to the individual SAFBDG “Use fats sparingly. Choose vegetable oils rather than hard fats” was low, breast cancer cases (52·5 %) were more likely to adhere to the total fat sub-recommendation (total fat >20 % and <30 % of total energy intake) than controls (45·2 %).

Table 4 provides results on the association between overall adherence to the SAFBDG, using data-driven tertiles for each SAFBDG recommendation, and the association with breast cancer risk. After adjusting for potential confounders, higher adherence (>5·0) *v.* lowest adherence (≤3·5) to the SAFBDG showed a significant inverse association with breast cancer risk overall (OR = 0·56, 95 % CI (0·38, 0·85), *P* = 0·006), among postmenopausal women (OR = 0·64, 95 % CI (0·40, 0·97), *P* = 0·034) as well as in oestrogen receptor positive (ER+) breast cancer (OR = 0·51, 95 % CI (0·32, 0·89), *P* = 0·004). No significant association with breast cancer risk was observed in premenopausal or obese women.

**Table 4:** The association between overall South African food based dietary guideline (SAFBDG) adherence and breast cancer risk, using data-driven tertiles (33rd and 66th percentiles)

	Adherence score	Cases		Control		Crude output		Adjusted model 2		|| *P*-value for stratification interactions
		*n*	%	*n*	%	OR	95 % CI	OR	95 % CI	
Overall (cases *n* 396; controls *n* 396)	≤3·5	188	47·5	153	38·6	1		1		
	>3·5; ≤5·0	117	29·5	120	30·3	0·84	0·60, 1·16	0·81	0·57, 1·16	n/a
	>5·0	91	23·0	123	31·1	0·58	0·39, 0·85	0·56	0·38, 0·85	n/a
	§ *P*_trend_					0·005		0·006		
*,†Premenopausal (*n* 267) (cases *n* 133; controls *n* 134)	≤3·5	58	43·6	50	37·3	1		1		
	>3·5; ≤5·0	45	33·8	43	32·1	0·84	0·45, 1·57	0·89	0·49, 1·63	0·880
	>5·0	30	22·6	41	30·6	0·53	0·25, 1·12	0·71	0·37, 1·36	0·782
	§ *P*_trend_					0·095		0·303		
*,†Postmenopausal (*n* 505) (cases *n* 248; controls n 257)	≤3·5	122	49·2	103	40·1	1		1		
	>3·5; ≤5·0	69	27·8	75	29·2	0·72	0·51, 1·20	0·75	0·48, 1·16	0·880
	>5·0	57	23·0	79	30·7	0·54	0·33, 0·88	0·64	0·40, 0·97	0·782
	§ *P*_trend_					0·014		0·034		
ER+ (*n* 298)	≤3·5	95	31·9	–		1		1		
	>3·5; ≤5·0	133	44·6	–		0·66	0·31, 1·44	0·75	0·49, 1·13	0·753
	>5·0	70	23·5	–		0·78	0·34, 1·79	0·51	0·32, 0·8	0·516
	§ *P*_trend_					0·563		0·004		
PR+ (*n* 263)	≤3·5	95	36·1	–		1		1		
	>3·5; ≤5·0	109	41·4	–		0·54	0·27, 1·10	0·82	0·53, 1·27	0·883
	>5·0	59	22·4	–		0·38	0·18, 0·78	0·65	0·40, 1·05	0·782
	§ *P*_trend_					0·008		0·077		
*,‡Obese (*n* 466) (cases = 231; controls = 235)	≤3·5	108	46·8	93	39·6	1		1		
	>3·5; ≤ 5·0	70	30·3	78	33·2	1·02	0·60, 1·75	0·72	0·46, 1·15	0·874
	>5·0	53	22·9	64	27·2	0·85	0·43, 1·66	0·74	0·45, 1·22	0·678
	§ *P*_trend_					0·624		0·239		

ER+, estrogen receptor positive; PR+, progesterone receptor positive.

* Unconditional logistic regression.

† Twenty missing values for menopausal status (fifteen cases and five controls).

‡ Obesity defined as BMI ≥ 30 kg/m^2^.

§ Indicating significance for OR to determine the association with breast cancer risk (trend analysis comparing highest *v*. lowest tertiles).

|| Indicating significance for stratification interactions.

Adjusted Model 2: Adjusted for ethnicity, total energy intake, alcohol intake, individual income per month, waist circumference (not adjusted for waist circumference when stratified by obesity status) and menopausal status (not adjusted for menopausal status when stratified by menopausal status).

The association between higher adherence to individual SAFBDG recommendations, using data-driven tertiles for each individual SAFBDG recommendation, with breast cancer risk is presented in Table 5. After adjustment for potential confounding factors, higher adherence to the recommendation “Enjoy a variety of foods” (measured by a dietary diversity score) showed an inverse association with breast cancer risk overall (OR = 0·46, 95 % CI (0·29, 0·70), *P* < 0·001), in postmenopausal women (OR = 0·48, 95 % CI (0·30, 0·77), *P* = 0·003) and in participants with ER+ and progesterone receptor positive (PR+) breast cancers (OR = 0·42, 95 % CI (0·26, 0·68), *P* < 0·001 and OR = 0·51, 95 % CI (0·31, 0·85), *P* = 0·010, respectively). With regard to the recommendation “Make starchy food part of most meals”, higher adherence showed an inverse association with ER+ breast cancer (OR = 0·65, 95 % CI (0·42, 0·99), *P* = 0·047). Furthermore, higher adherence to the recommendation “Eat plenty of vegetables and fruit everyday” showed an inverse association with breast cancer risk overall (OR = 0·58, 95 % CI (0·38, 0·86), *P* = 0·008), in postmenopausal women (OR = 0·62, 95 % CI (0·39, 0·99), *P* = 0·046) and in participants with ER+ and PR+ breast cancers (OR = 0·51, 95 % CI (0·32, 0·81), *P* = 0·005 and OR = 0·59, 95 % CI (0·37, 0·94), *P* = 0·028, respectively). Higher consumption of milk, maas or yoghurt showed an inverse association with breast cancer risk overall (OR = 0·69, 95 % CI (0·47, 0·97), *P* = 0·025), in postmenopausal women (OR = 0·69, 95 % CI (0·43, 0·98), *P* = 0·039) and in participants with ER+ breast cancers (OR = 0·54, 95 % CI (0·35, 0·84), *P* = 0·006). Higher adherence to the recommendation “Fish, chicken, lean meat or eggs can be eaten daily” showed an inverse association with breast cancer risk overall (OR = 0·67, 95 % CI (0·46, 0·95), *P* = 0·036) and in participants with ER+ breast cancer (OR = 0·56, 95 % CI (0·36, 0·87), *P* = 0·010).

**Table 5:** The association between adherence to individual South African food based dietary guideline (SAFBDG) recommendations and breast cancer risk, using data-driven tertiles (33rd and 66th percentiles)

South African food based dietary guidelines	Score adherence	Overall* (cases *n* 396; controls *n* 396)		**,‡Premenopausal (cases = 133; controls = 134)		**,‡Postmenopausal (cases = 248; controls = 257)		†ER+ (*n* 298)		†PR+ (*n* 263		*,§Obese (cases = 231; controls = 235)	
		OR	95 % CI	OR	95 % CI	OR	95 % CI	OR	95 % CI	OR	95 % CI	OR	95 % CI
Enjoy a variety of foods	0	1		1		1		1		1		1	
	0·5	0·84	0·57, 1·23	0·73	0·40, 1·32	0·90	0·57, 1·44	0·83	0·54, 1·26	0·81	0·52, 1·25	1·02	0·64, 1·64
	1	0·46	0·29, 0·70	0·60	0·30, 1·19	0·48	0·30, 0·77	0·42	0·26, 0·68	0·51	0·31, 0·85	0·63	0·38, 1·04
	*P*_trend_	<0·001		0·147		0·003		<0·001		0·010		0·069	
Be active	0	1		1		1		1		1		1	
	0·5	0·94	0·61, 1·45	0·58	0·27, 1·28	1·20	0·73, 1·95	0·81	0·40, 1·33	0·84	0·50, 1·43	0·88	0·52, 1·49
	1	0·91	0·48, 1·72	0·56	0·18, 1·80	1·23	0·64, 2·34	0·77	0·37, 1·63	0·82	0·37, 1·81	1·27	0·65, 2·48
	*P*_trend_	0·771		0·332		0·531		0·501		0·620		0·485	
Make starchy food part of most meals.	0	1		1		1		1		1		1	
	0·5	1·00	0·69, 1·46	1·21	0·64, 2·32	0·89	0·58, 1·35	0·94	0·62, 1·44	1·09	0·71, 1·71	0·96	0·62, 1·50
	1	0·86	0·59, 1·26	1·07	0·56, 2·07	0·80	0·50, 1·29	0·65	0·42, 0·99	0·84	0·53, 1·33	0·79	0·47, 1·30
	*P*_trend_	0·445		0·832		0·363		0·047		0·451		0·354	
Eat plenty of vegetables and fruit every day	0	1		1		1		1		1		1	
	0·5	1·03	0·71, 1·46	0·73	0·40, 1·32	1·25	0·81, 1·93	1·11	0·75, 1·67	1·06	0·70, 1·59	1·14	0·73, 1·78
	1	0·58	0·38, 0·86	0·60	0·30, 1·19	0·62	0·39, 0·99	0·51	0·32, 0·81	0·59	0·37, 0·94	0·77	0·47, 1·26
	*P*_trend_	0·008		0·089		0·046		0·005		0·028		0·291	
Eat dry beans, split peas, lentils and soya regularly	0	1		1		1		1		1		1	
	0·5	0·83	0·53, 1·30	1·58	0·68, 3·64	0·64	0·38, 1·08	0·83	0·51, 1·37	0·90	0·52, 1·56	0·75	0·43, 1·31
	1	0·82	0·59, 1·15	1·04	0·60, 1·80	0·83	0·55, 1·26	0·95	0·65, 1·37	0·96	0·65, 1·41	0·69	0·45, 1·06
	*P*_trend_	0·255		0·889		0·390		0·774		0·821		0·088	
Have milk, maas or yoghurt every day	0	1		1		1		1		1		1	
	0·5	0·78	0·54, 1·13	0·87	0·47, 1·60	0·79	0·52, 1·23	0·74	0·49, 1·13	0·93	0·59, 1·47	1·06	0·68, 1·68
	1	0·69	0·47, 0·97	0·75	0·39, 1·44	0·69	0·43, 0·98	0·54	0·35, 0·84	0·66	0·41, 1·05	0·78	0·48, 1·23
	*P*trend	0·025		0·392		0·039		0·006		0·080		0·319	
Fish, chicken, lean meat or eggs can be eaten daily	0	1		1		1		1		1		1	
	0·5	0·82	0·58, 1·16	0·64	0·35, 1·17	0·96	0·63, 1·45	0·74	0·49, 1·11	0·85	0·56, 1·28	0·88	0·57, 1·37
	1	0·67	0·46, 0·95	0·72	0·36, 1·45	0·74	0·46, 1·20	0·56	0·36, 0·87	0·70	0·44, 1·11	0·93	0·56, 1·54
	*P*_trend_	0·036		0·355		0·223		0·010		0·133		0·773	
Use fats sparingly – choose vegetable oils, rather than hard fats: total fat‖	0·5	1		1		1		1		1		1	
	0·25	0·79	0·55, 1·15	0·73	0·40, 1·33	0·84	0·53, 1·34	0·76	0·51, 1·14	0·77	0·50, 1·19	0·76	0·48, 1·21
	0	1·05	0·71, 1·54	1·41	0·72, 2·76	0·92	0·59, 1·44	0·96	0·62, 1·49	0·97	0·62, 1·55	1·10	0·68, 1·77
	*P*_trend_	0·825		0·314		0·717		0·871		0·919		0·697	
Use fats sparingly – choose vegetable oils, rather than hard fats: total saturated fat‖	0·5	1		1		1		1		1		1	
	0·25	0·99	0·68, 1·46	1·28	0·70, 2·35	0·84	0·53, 1·34	1·00	0·66, 1·52	0·97	0·62, 1·52	0·90	0·56, 1·45
	0	1·34	0·92, 1·97	1·26	0·67, 2·38	1·29	0·82, 2·04	1·26	0·82, 1·93	1·15	0·74, 1·81	1·30	0·82, 2·07
	*P*_trend_	0·132		0·476		0·265		0·285		0·534		0·269	
Use sugar and foods and drinks high in sugar sparingly	1	1		1		1		1		1		1	
	0·5	1·06	0·74, 1·52	1·21	0·65, 2·25	1·10	0·72, 1·70	0·99	0·66, 1·49	1·03	0·67, 1·58	1·28	0·81, 2·01
	0	1·01	0·70, 1·46	1·43	0·74, 2·73	0·90	0·58, 1·39	0·96	0·63, 1·43	1·07	0·70, 1·64	0·94	0·58, 1·50
	*P*_trend_	0·932		0·285		0·626		0·832		0·765		0·784	

ER+, estrogen receptor positive; PR+, Progesterone receptor positive.

* Adjusted for total energy intake, alcohol intake, individual income/month, ethnicity, waist circumference, physical activity, (unless the variable was part of the recommendation under investigation) and menopausal status (not when stratified by menopausal status).

† Stratified by estrogen receptor status or progesterone receptor status.

‡ Twenty missing values for menopausal status (fifteen cases and five controls).

§ Stratified by obesity defined as BMI ≥ 30 kg/m^2^ and using unconditional logistic regression.

‖ Adherence to the guideline “Use fats sparingly. Choose vegetable oils, rather than hard fats” are measured by two subcategories, total saturated fat and total saturated fat intake.

** Stratified by menopausal status or obesity and using unconditional logistic regression.

0 indicates the lowest and 1 the highest adherence to the specific recommendation.

No significant *P*-values were observed when interactions among strata were assessed.

## Discussion

To our knowledge, this is the first study investigating the relationship between adherence to the SAFBDG and breast cancer risk. The results indicate that higher adherence to the SAFBDG, using data-driven cut-points, may reduce the risk of developing breast cancer in this population overall, in postmenopausal women and for women with ER+ breast cancer. The strongest inverse associations with breast cancer risk were seen for higher adherence to the following individual SAFBDG recommendations “Enjoy a variety of foods”, “Eat plenty of fruit and vegetables every day”, “Have milk, maas or yoghurt every day” and “Fish, chicken, lean meat or eggs can be eaten daily”. The study results further indicate a concerning low level of adherence to the SAFBDG, whether adherence was measured as overall adherence or individual recommendation adherence.

Higher adherence to the SAFBG (measured with data-driven cut-points) may protect against the development of breast cancer in this population. Studies investigating the association between adherence to the SAFBDG (and other dietary guidelines) and nutrition-related diseases in this South African population are still limited. However, our findings are in line with results from international studies investigating adherence to national Food Based Dietary Guidelines in association with non-communicable disease and obesity risk. Although national Food Based Dietary Guidelines may differ, based on the population, Food Based Dietary Guidelines generally promote similar healthy eating behaviour/patterns globally. A Danish Cohort study of 54 305 participants showed that higher adherence to the Danish Food Based Dietary Guidelines had an inverse association with Type 2 diabetes and CVD^(33)^. A Dutch Cohort study showed that higher adherence to the Dutch Food Based Dietary Guidelines was inversely associated with overall mortality and non-communicable diseases such as Type 2 diabetes and colorectal cancer^(34)^. The European Prospective Investigation into Cancer and Nutrition study (EPIC-Granada study conducted in Spain) showed that higher adherence to the Spanish Dietary Guidelines was associated with a lower risk of being obese^(35)^. These global findings highlight the importance of adhering to national Food Based Dietary Guidelines to reduce non-communicable diseases such as breast cancer.

### Associations and adherence to individual South African food based dietary guideline recommendations

#### “Enjoy a variety of foods”

Higher adherence to the individual SAFBDG recommendation “Enjoy a variety of foods”, using data-driven cut-points, showed an inverse association with breast cancer risk. This indicate that consumption of a variety of foods may play an important role in breast cancer prevention. However, more than 60 % of cases and nearly half of controls did not adhere to this recommendation when suggested adherence cut-points were used. Similar results from the South African National Health and Nutrition Examination Survey showed that 50 % of the black South African population had a DDS below four^(31)^. This is a concern as the low DDS in the current study indicates that a variety of foods and thus a variety of micronutrients, which may protect against breast cancer, are not consumed^(3,31)^.

#### “Eat plenty of fruit and vegetables every day” and “eat dry beans, split peas, lentils and soya regularly”

Higher adherence to the individual SAFBDG recommendation “Eat plenty of fruit and vegetables every day”, using data-driven cut-points, was inversely associated with breast cancer risk in the current study. Worrisome, however, is that adherence to the SAFBDG recommendation “Eat plenty of fruit and vegetables every day” (using suggested adherence cut-points) was low in both cases and controls in our study. The Prospective Urban and Rural Epidemiology cohort study, conducted in the North-West Province of South Africa, showed that only 31·5 % of urban women (*n* 355) adhered to this recommendation^(29)^. Low fruit and vegetable consumption was also observed in other South African regions^(21,36)^. In addition, concerning low levels of adherence to the individual SAFBDG recommendation “Eat dry beans, split peas, lentils and soya regularly” was observed when suggested adherence cut-points were used in the analysis and is in line with other studies reporting low legume consumption in South Africa^(29)^. Low adherence to these respective recommendations is of concern as high fibre foods such as fruit, vegetables and legumes have been linked to a decreased risk of colorectal cancer and can help maintain a healthy weight necessary to decrease the risk for postmenopausal breast cancer^(3)^.

#### “Having milk, maas or yoghurt every day and “fish, chicken, lean meats or eggs can be eaten daily”

Higher adherence to the individual SAFBDG recommendations “Having milk, maas or yoghurt every day and “Fish, chicken, lean meats or eggs can be eaten daily” showed inverse associations with breast cancer risk when data-driven cut-points were used. However, both cases and controls showed worrying low levels of adherence to the SAFBDG recommendation “Have milk, maas or yoghurt everyday” (using suggested adherence cut-points). The Prospective Urban and Rural Epidemiologica study, mentioned above, showed similar low adherence levels (11·5 %) to this recommendation amongst urban women (400 g of milk, maas or yoghurt or 50 g hard cheese/d)^(29)^. Low adherence to this recommendation is concerning since the evidence suggests that dairy products and diets high in Ca may decrease the risk of premenopausal breast cancer^(3)^.

#### “Make starchy foods part of most meals”

Almost 60 % of both cases and controls did not adhere to the SAFBDG recommendation “Make starchy foods part of most meals” when suggested adherence cut-points were used. This finding was unexpected since staple foods, such as fortified maize meal, bread, rice, etc., promoted in this recommendation, cost less per unit of energy than animal products, fruit and vegetables and is therefore considered affordable^(37)^. Despite the low consumption of minimally processed starchy foods, total carbohydrate intake for both cases (61·4 %) and controls (64·0 %) was within the recommended macronutrient distribution range (45–65 % of total energy intake). This finding indicates that not all starchy foods consumed are of high nutritional value and reflect the changes in dietary carbohydrate consumption, from high fibre and nutrient dense starchy staple food intakes to higher consumption of refined starches, lacking nutrients and having a high added sugar content.

#### “Use fats sparingly. Choose vegetable oils rather than hard fats” and “use sugar and foods and drinks high in sugar sparingly”

Adherence to the SAFBDG recommendations: (1) “Use fats sparingly. Choose vegetable oils rather than hard fats” and (2) “Use sugar & foods & drinks sparingly” also showed low levels of adherence in both cases and controls when suggested portions were used as adherence cut-points. Similar, low levels of adherence to the SAFBDG recommendation regarding total fat intake were observed in Cape Town, South Africa^(38)^. With regard to added sugar intake, a review of dietary surveys in the adult South African population from 2000 to 2015 and cross-sectional studies showed that added sugar intake was greater than the recommended 10 % of total energy intake in various provinces of South Africa (North-West, KwaZulu-Natal, Western Cape, Free state)^(1,38)^. Diets high in saturated fat and added sugar are concerning because diets high in saturated fat and added sugar have been linked to a higher risk of being overweight or obese, which is a known risk factor for postmenopausal breast cancer^(4)^.

#### “Be active”

Apart from low adherence to the nutrition-related SAFDG recommendations, adherence to the SAFBDG recommendation “Be active” also showed worrying low adherence levels in both breast cancer cases and controls in the current study. This low physical activity level is not a new finding and is in line with several studies conducted amongst black South African women^(39,40)^. The finding is alarming as physical activity may protect against breast cancer development and being overweight or obese^(3)^.

### Level of adherence to the overall South African food based dietary guidelines

A modelling study, reviewing global adherence to national food based dietary guidelines in 85 countries, showed that South Africa is among the countries with the lowest adherence to the national food based dietary guidelines^(41)^. In line with the above review, the results of our study clearly showed low adherence to the overall and individual SAFBDG recommendations.

Poverty, influencing purchasing power, and a growing obesogenic food environment are considered key barriers to healthier eating patterns in South Africa^(37)^. Additionally, the Strategy for the Prevention and Control of Obesity in South Africa states that high crime rates and gender-based violence, especially in urban South African areas, contribute to the perception of it being unsafe to exercise outdoors and could also contribute to low physical activity levels (all ethnicities)^(42)^. A lack of exercise space due to small plots and size of physical buildings in low-income households may also be considered a potential limitation to physical activity^(43)^. Also, the many challenges associated with measuring self-reported physical activity levels in epidemiological studies such as recall bias of participants may further contribute to low physical activity levels. Furthermore, a lack of knowledge (especially in resource poor settings), education and skills regarding food preparation methods (especially for beans/lentils); length of preparation time; taste preferences (unsweetened products *v*. sweetened products) and perceptions towards a healthy diet and social and cultural influences may also contribute to the low adherence levels observed^(1)^. Given the effect of the COVID-19 pandemic, resulting in a higher unemployment rate and higher food prices, adherence to the SAFBDG may decrease even further^(44)^. Low adherence to the recommendation “Eat dry beans, split peas, lentils and soya regularly” is not likely to be influenced by economic status since beans and other legumes are affordable in South Africa^(37)^. However, the cost occurred as a result of the length of preparation of beans and other legumes may influence the consumption thereof. Therefore, more research is required to understand the drivers of consumption of specific food groups such as beans and other legumes in this population.

Results of our study suggest that dietary intake that is not well aligned with the SAFBDG recommendations is associated with an increased risk of developing breast cancer in this black female population of Soweto, South Africa. It is therefore critical to also create sustainable and healthy food environments that support the affordability, availability and accessibility of healthy foods and safe environments that support physical activity in order to enable adherence to the SAFBDG in this population. However, creating sustainable and healthy food environments in South Africa is not an easy task and requires multi-sectoral and transdisciplinary public health engagement, beyond those already in place^(45)^.

Furthermore, results of our study are in line with a recent study, conducted in the same black female population of Soweto, South Africa, which investigated the association between adherence to the 2018 WCRF/AICR Cancer Prevention Recommendations and breast cancer risk^(10)^. Both sets of recommendations showed that higher consumption of fruit and vegetables may reduce breast cancer risk in this population. The 2018 WCRF/AICR Cancer Prevention Recommendations emphasised the importance of following an overall healthy lifestyle (being physically active, a healthy weight and following healthy diet) for breast cancer prevention. Results of the current study complement these findings of Jacobs and colleagues (2021) by (1) highlighting the potential benefits of specific foods (milk, maas, yoghurt, lean meats and eggs) and (2) emphasising the importance of following a diverse diet for breast cancer prevention in this population. While the findings of both studies provide valuable insights in the lifestyle and diets of black women from Soweto, more research is required to understand how the 2018 WCRF/AICR Cancer Prevention Recommendations and the SAFBDG could be used as key nutrition intervention/tool in breast cancer prevention studies.

Strengths of the current study include the fact that cases were recruited prior to any breast cancer treatment and that the questionnaires used to obtain data were proven to be validated and data used in the analysis were standardised and administered by trained personnel. Limitations include the relatively limited sample size of the current study, the nature of the case–control study design which is prone to differential biases of cases and the use of a QFFQ to collect dietary data which relies on the memory of participants and is therefore more prone to recall bias. Dietary intake and physical activity were measured over the past month when habitual dietary intake of case participants could have changed due to illness and may contribute to random misclassification and under estimation of dietary intake. In addition, although dietary intakes were captured throughout the year (in different participants) and that breast cancer cases and controls were recruited little time apart, seasonal variability of foods (not adjusted for) may have influenced usual reporting of dietary intakes. It is also noteworthy that QFFQ are not ideal for measuring absolute dietary intakes as their main goal is to measure relative dietary intakes, allowing the ranking of people according to their dietary intakes. Therefore, comparison with fixed cutoffs is not recommended, especially in a population with highly skew data, which motived the use of data-driven tertiles for dividing the participants according to their dietary intakes and to measure adherence to the SAFBDG. Using such an alternative method may have influenced the overall results.

The methodology used in the current study is one of the first attempts to measure quantitative adherence to the SAFBDG and requires more investigation to establish a standardised adherence algorithm. We followed the exact recommendations as stated in the technical support papers of the revised SAFBDG. But, precise recommendations were not always stated such as in the case of measuring adherence to the recommendations “Make starchy foods part of most meals” and “Eat dry beans, split peas, lentils and soya regularly”. To measure adherence to these two recommendations, assumptions were made, based on previous studies in South Africa or consulting with nutrition experts in South Africa. Such assumptions made the operationalisation of the current study challenging.

In conclusion, higher adherence to the SAFBDG showed inverse associations with breast cancer risk overall, in postmenopausal women and for women with ER+ breast cancer. In particular, the increased consumption of a variety of foods, fruit and vegetables, milk, maas or yoghurt and lean meat or eggs showed strong inverse associations with breast cancer risk. However, the black female population included in the current study showed concerning low levels of adherence to the SAFBDG. It is therefore necessary to promote adherence to the SAFBDG in both preventative education campaigns/actions and the creation of sustainable and healthy food environments that enhances the affordability, availability and accessibility of healthier foods, together with safe environments that support increased physical activity in order to enable adherence to the SAFBDG.

## Supplementary material

For supplementary material accompanying this paper visit https://doi.org/10.1017/S1368980021004675. click here to view supplementary material
