# Supplementary material for: Adherence to the South African food based dietary guidelines may reduce breast cancer risk in black South African women: the South African Breast Cancer (SABC) study
Source: Public Health Nutr. 2021 Nov 29;25(10):2805–21. doi: 10.1017/S1368980021004675 (PMC9991845; doi:10.1017/S1368980021004675)
Supplement: Supplementary file 1 [file S1368980021004675sup.zip › S1368980021004675sup001.docx]

**Supplementary Table 1 Distribution of adherence to the individual SAFBDGs between breast cancer cases and controls, using data driven tertiles (33^rd^ and 66^th^ percentiles) to assess the association with breast cancer risk.**

| **South African Food Based Dietary Guideline** | **Operationalization** | **Score contribution** | | **Cases n=396**  n (%) | | **Controls n=396**  n (%) | | | **P-value overall** | | |
| --- | --- | --- | --- | --- | --- | --- | --- | --- | --- | --- | --- |
| 1) Enjoy a variety of foods. | Dietary diversity score <3 | 0 | | 205 (52) | | 165 (42) | | | <0.001 | | |
|  | Dietary diversity score ≥3 & <4.5 | 0.5 | | 118 (30) | | 108 (27) | | |  |  |  |
|  | Dietary diversity score ≥4.5 | 1 | | 73 (18) | | 123 (31) | | |  |  |  |
| 2) Be active. | Total physical activity <49 min/week | 0 | | 120 (30) | | 132 (33) | | | 0.527 | | |
|  | Total physical activity ≥49 min & <65 min/week | 0.5 | | 128 (32) | | 130 (33) | | |  |  |  |
|  | Total physical activity ≥65 min/week | 1 | | 148 (38) | | 134 (34) | | |  |  |  |
| 3) *Make starchy foods part of most meals. | Starch exchanges ≤7/day | 0 | | 157 (40) | 132 (33) | | 0.182 | | |  |  |
|  | Starch exchanges <11/day & >7/day | 0.5 | | 123 (31) | 135 (34) | |  | | |  |  |
|  | Starch exchanges ≥11/day | 1 | | 116 (29) | 129 (33) | |  | | |  |  |
| 4) Eat plenty of vegetables and fruit every day. | Fruit & vegetables <255 g/day | 0 | | 152 (38) | | 131 (33) | | | 0.001 | | |
|  | Fruit & vegetables ≥255 g & <753 g/day | 0.5 | | 156 (39) | | 131 (33) | | |  |  |  |
|  | Fruit & vegetables ≥753 g/day | 1 | | 88 (23) | | 134 (34) | | |  |  |  |
| 5) Eat dry beans, split peas, lentils and soya regularly. | Legumes 0 g/day | 0 | | 228 (58) | | 215 (54) | | | 0.632 | | |
|  | Legumes ≤6 g/day | 0.5 | | 52 (13) | | 54 (14) | | |  |  |  |
|  | Legumes >6 g/day | 1 | | 116 (29) | | 127 (32) | | |  |  |  |
| 6) Have milk, maas or yoghurt every day. | Milk or maas or yoghurt <67 ml/day | 0 | | 161 (41) | | 131 (33) | | | 0.046 | | |
|  | Milk or maas or yoghurt ≥67 ml & <129 ml/day | 0.5 | | 128 (32) | | 131 (33) | | |  |  |  |
|  | Milk or maas or yoghurt ≥129 ml/day | 1 | | 107 (27) | | 134 (34) | | |  |  |  |
| 7) Fish, chicken, lean meat or eggs can be eaten daily. | Lean meat/fish <5.7 g or <9.7 g eggs/day | 0 | | 158 (40) | | 131 (33) | | | 0.062 | | |
|  | Lean meat/fish ≥5.7 g or ≥9.7 g eggs/day | 0.5 | | 148 (37) | | 150 (38) | | |  |  |  |
|  | Lean meat/fish ≥35.7 g or ≥57.1 g eggs/day | 1 | | 90 (23) | | 115 (29) | | |  |  |  |
| 8) Drink lots of clean, safe water. | Guideline not considered in this study | |  |  |  |  |  |  |  |  |  |
| 9) †Use fats sparingly. Choose vegetable oils, rather than hard fats. | Total fat ≥24% & <30% of TEI | 0.5 | | 113 (29) | | 131 (33) | | | 0.362 | | |
|  | Total fat <24% of TEI | 0.25 | | 144 (36) | | 131 (33) | | |  |  |  |
|  | Total fat ≥30% of TEI | 0 | | 139 (35) | | 134 (34) | | |  |  |  |
|  | Saturated fat <7% of TEI | 0.5 | | 154 (39) | | 131 (33) | | | 0.441 | | |
|  | Saturated fat ≥7% & <9% of TEI | 0.25 | | 120 (30) | | 131 (33) | | |  |  |  |
|  | Saturated fat ≥9% of TEI | 0 | | 122 (31) | | 134 (34) | | |  |  |  |
| 10) Use sugar and foods and drinks high in sugar sparingly. | Added sugar <9% of TEI | 1 | | 123 (31) | | 131 (33) | | | 0.618 | | |
|  | Added sugar ≥9% & <16% of TEI | 0.5 | | 144 (36) | | 131 (33) | | |  |  |  |
|  | Added sugar ≥16% of TEI | 0 | | 129 (33) | | 134 (34) | | |  |  |  |
| 11) Use salt and food and drinks high in salt sparingly. | Guideline not considered in this study | | | | | | |  | | |  |

TEI: total energy intake;
*No specific indication of portion size or frequency of consumption by the SAFBDGs. General guideline is to consume 10 starchy food guide units per day (based on 8 500 kJ intake/day) ^(29)^ (one food guide unit: maize meal porridge, soft/maltabella/oats= 125 g, maize meal porridge, stiff=60 g, crumbly=45g bread=35 g, potatoes/sweet potatoes= 100 g, cooked pasta/samp/whole grains=75 g, unsweetened breakfast cereals 25 g, and cooked rice=65 g) of the study population, ^(70, 71)^.
†Adherence to the guideline “Use fats sparingly. Choose vegetable oils, rather than hard fats” are measured by two subcategories, total fat and total saturated fat intake
